# Otitis Media Practice During the COVID-19 Pandemic

**DOI:** 10.3389/fcimb.2021.749911

**Published:** 2022-01-07

**Authors:** Tal Marom, Jacob Pitaro, Udayan K. Shah, Sara Torretta, Paola Marchisio, Ayan T. Kumar, Patrick C. Barth, Sharon Ovnat Tamir

**Affiliations:** ^1^ Department of Otolaryngology-Head and Neck Surgery, Samson Assuta Ashdod University Hospital, Ben Gurion University Faculty of Health Sciences, Ashdod, Israel; ^2^ Department of Otolaryngology-Head and Neck Surgery, Shamir Medical Center (formerly Assaf Harofeh Medical Center), Zerifin, Israel, affiliated to the Sackler Faculty of Medicine, Tel Aviv University, Tel Aviv, Israel; ^3^ Pediatric Otolaryngology, Delaware Valley, and Enterprise Chief of Credentialing, Nemours Children’s Health System, Wilmington, DE, United States; ^4^ Departments of Otolaryngology-Head & Neck Surgery and Pediatrics, Sidney Kimmel Medical College, Thomas Jefferson University, Philadelphia, PA, United States; ^5^ Fondazione IRCCS Ca’ Granda Ospedale Maggiore Policlinico, Milan, Italy; ^6^ Department of Clinical Sciences and Community Health, University of Milan, Milan, Italy; ^7^ Department of Clinical Sciences and Community Health, Fondazione IRCCS Ca’ Granda Ospedale Maggiore Policlinico, Milan, Italy; ^8^ Department of Pathophysiology and Transplantation, University of Milan, Milan, Italy; ^9^ Department of Otolaryngology-Head & Neck Surgery, Sidney Kimmel Medical College, Thomas Jefferson University, Philadelphia, PA, United States; ^10^ Pediatric Otolaryngology, Delaware Valley Nemours Children’s Health System, Wilmington, DE, United States

**Keywords:** otitis media, COVID-19, coronavirus infection, admission, burden analysis, acute otitis media, otitis media with effusion, mastoiditis

## Abstract

The global coronavirus disease-2019 (COVID-19) pandemic has changed the prevalence and management of many pediatric infectious diseases, including acute otitis media (AOM). Coronaviruses are a group of RNA viruses that cause respiratory tract infections in humans. Before the COVID-19 pandemic, coronavirus serotypes OC43, 229E, HKU1, and NL63 were infrequently detected in middle ear fluid (MEF) specimens and nasopharyngeal aspirates in children with AOM during the 1990s and 2000s and were associated with a mild course of the disease. At times when CoV was detected in OM cases, the overall viral load was relatively low. The new severe acute respiratory syndrome coronavirus-2 (SARS-CoV-2) is the causative pathogen responsible for the eruption of the COVID-19 global pandemic. Following the pandemic declaration in many countries and by the World Health Organization in March 2020, preventive proactive measures were imposed to limit COVID-19. These included social distancing; lockdowns; closure of workplaces; kindergartens and schools; increased hygiene; use of antiseptics and alcohol-based gels; frequent temperature measurements and wearing masks. These measures were not the only ones taken, as hospitals and clinics tried to minimize treating non-urgent medical referrals such as OM, and elective surgical procedures were canceled, such as ventilating tube insertion (VTI). These changes and regulations altered the way OM is practiced during the COVID-19 pandemic. Advents in technology allowed a vast use of telemedicine technologies for OM, however, the accuracy of AOM diagnosis in those encounters was in doubt, and antibiotic prescription rates were still reported to be high. There was an overall decrease in AOM episodes and admissions rates and with high spontaneous resolution rates of MEF in children, and a reduction in VTI surgeries. Despite an initial fear regarding viral shedding during myringotomy, the procedure was shown to be safe. Special draping techniques for otologic surgery were suggested. Other aspects of OM practice included the presentation of adult patients with AOM who tested positive for SARS-2-CoV and its detection in MEF samples in living patients and in the mucosa of the middle ear and mastoid in post-mortem specimens.

## Coronavirus Family

The coronavirus (CoV) family is a group of enveloped, positive-sense, single-stranded, highly diverse RNA viruses, with sizes ranging from 60-140 nm in diameter, having crown shape projections on their surface, hence their name: coronaviruses. Four main serogroups exist HKU1, NL63, 229E, and OC43, which have previously been shown to affect humans, and usually caused mild upper respiratory tract infections (URTIs) (Zumla et al., 2016). CoV was also detected from nasopharyngeal aspirates (NPAs) in infants during asymptomatic health visits (Chonmaitree et al., 2015). Research interest in the CoV family has been minimal over the past few decades because RNA viruses were hard to study, and large sero-epidemiolgical surveys conducted during the 1970s and 1980s showed high antibody titers in both children and adults, assuming that CoV infections were common and self-limiting, like any other respiratory viruses (Wenzel et al., 1974; Sarateanu and Ehrengut, 1980; Schmidt et al., 1986).

Over the last two decades, 3 new CoV members emerged: 1) Severe Acute Respiratory Syndrome-associated coronavirus (*SARS-CoV*), identified in 2002 in China, which caused a limited-scale epidemic involving 2 dozen countries with ~8000 cases and ~800 deaths (fatality rate: 9.6%) (Drosten et al., 2003); 2) Middle East Respiratory Syndrome-associated coronavirus (*MERS-CoV*), identified in 2012 in Saudi Arabia, which affected ~2,500 patients and caused ~800 deaths (fatality rate: 35%) (Rasmussen et al., 2016), and 3) *SARS-CoV-2*, identified in China in 2019, which caused a global pandemic, commonly known as coronavirus disease-2019 (*COVID-19*), currently affecting >191 millions of patients and causing >4.1 million deaths worldwide (source: Johns Hopkins University Center for Systems Science and Engineering, https://github.com/CSSEGISandData/COVID-19, accessed July 20, 2021). 

## Evidence for CoV Detection in Otitis Media Cases

### Old CoV Serotypes

**Table 1** shows published reports on CoV detection in pediatric AOM cases. Using traditional polymerase chain reaction (PCR) and microplate hybridization assays, CoV detection rate in middle ear fluid (MEF) and NPAs in AOM cases was anecdotal. However, when newer real-time PCR assays became available and in widespread use, CoV detection rates increased up to 50% (Chonmaitree et al., 2008). A recent review showed that CoV load in the middle ear in various OM cases was overall very low (Liaw et al., 2021). There were no reports on AOM cases during the SARS-CoV and MERS outbreaks.

**Table 1:** CoV detection in AOM cases.

Country	Year	Age	No. of Children	Main Findings
Finland (Pitkaranta et al., 1998)	1998	3m-7y	69	CoV RNA was detected in both MEF and NPA in 5 (5%), in MEF alone in 2 (2%), and in NPA alone in 9 (10%). RSV and HCV were detected in 1 NPA sample.
Finland (Nokso-Koivisto et al., 2000)	2000	2-24m	329	In confirmed AOM cases: 13 NPA CoV+, but no CoV+ MEF specimens.
France (Vabret et al., 2005)	2005	<20y	300	Of the 28/300 patients that had NPA CoV+, 28% had AOM.
Turkey (Bulut et al., 2007)	2006	6-144m	120	5/42 pure viral cases had MEF CoV+.
Finland (Ruohola et al., 2006)	2006	7-71m	79	MEF collected from children with otorrhea from TTs: 1 *Moraxella catarrhalis* + rhinovirus + CoV and 1 *Streptococcus pneumoniae* + *Haemophilus influenzae* + CoV.
USA (Chonmaitree et al., 2008)	2008	6m-3y	294	50% CoV+ detection rate in 440 AOM episodes.
Australia (Wiertsema et al., 2011)	2011	6-36m	180	14.4% NPA and 4.9% MEF samples were CoV+.
The Netherlands (Stol et al., 2012)	2012	<5y	116	MEF collected from children during TT surgery: 4/116 (3%) were CoV+.

AOM, acute otitis media; MEF, middle ear fluid; NPA, nasopharyngeal aspirate; CoV, coronavirus; RSV, respiratory syncytial virus, TT, tympanostomy tube.

### SARS-2-CoV

The basis for the hypothesis that suggested SARS-2-CoV infection can spread from the nasopharynx to the middle ear can be explained by the abundant expression of angiotensin converting enzyme (ACE)-2 receptors by goblet cells lining the Eustachian tube mucosa, which are critical for the intracellular entry of SARS-CoV-2 (Matusiak and Schurch, 2020; McMillan et al., 2021).

Albeit the abundance of ACE-2 receptors in the Eustachian tube, only several reports were published linking SARS-2-CoV infection to AOM. The first description was a 35-year-old Turkish female who presented with otalgia and tinnitus. Otoscopy, audiometry, and tympanometry confirmed AOM. Her NPA was positive for SARS-CoV-2, but she had no typical respiratory symptoms (Fidan, 2020). Because myringotomy was not performed, it was impossible to determine SARS-CoV-2 presence in the MEF. A case-series publication described 8 Iranian adult COVID-19 patients with AOM (Raad et al., 2021). Interestingly, 1 patient tested negative for SARS-CoV-2 from the oropharynx, but his MEF sample, obtained *via* myringotomy, tested positive. A 23-year-old American COVID-19 patient presented with complicated AOM and facial palsy, although his MEF sample tested negative for SARS-CoV-2 (Mohan et al., 2021). SARS-CoV-2 was detected in the mastoid and middle ear mucosa in autopsies performed 14-46h post-mortem in 2/3 COVID-19 deceased adult patients who did not have a preceding AOM episode (Frazier et al., 2020). 

## Reduction in Otitis Media Burden

Following the declaration of the COVID-19 pandemic by the World Health Organization in early March 2020 (Jee, 2020), preventive proactive measures were imposed to limit SARS-CoV-2 transmission and infection, including social distancing, recommendations to stay at home, lockdowns in varying severities, closure of workplaces, kindergartens, and schools, increased hygiene measures, increased use of antiseptics and alcohol-based gels, frequent temperature measurements, and mask-wearing.

### Reduction in Overall Pediatric Emergency Department Visits

The COVID-19 pandemic and national and international interventions aimed at limiting its spread deeply changed “traditional” healthcare habits, limiting people’s mobility and access to medical facilities. Some Health and Welfare policy measures even discouraged non-urgent access to (pediatric) emergency departments (PED), and more generally any frontal visits to family physicians/pediatricians, which were not considered urgent. A substantial reduction in the number of pediatric referrals/admissions to PEDs has been globally reported in Singapore, USA, Italy, and Argentina (Chong et al., 2020; McBride et al., 2020; Ferrero et al., 2021; Finkelstein et al., 2021; Pepper et al., 2021).

### Reduction in Pediatric AOM/OME Cases

The measures adopted to contain the COVID-19 pandemic also resulted in a decrease of airborne-mediated respiratory infections other than COVID-19, including URTI, bronchiolitis, and AOM (Kuitunen et al., 2020; Lin et al., 2020; Torretta et al., 2020; Alde et al., 2021; Angoulvant et al., 2021; Torretta et al., 2021). In addition, the number of children presenting to healthcare facilities for non-urgent complaints, such as AOM or otitis media with effusion (OME), was also affected by the fear of contracting COVID-19 at the hospital or the outpatient clinics. Thus, there was a decrease in healthcare utilization for pediatric AOM/OME.

Reports from Milan, the largest and the most densely populated city in Lombardia, the Northern Italian region placed at the epicenter of the Italian epidemic, where all elective medical activities were discontinued between March-May 2020, showed a substantial reduction in pediatric OM burden (Alde et al., 2021; Torretta et al., 2021). All outpatient periodic visits for otitis-prone children regularly followed at a tertiary outpatient clinic were replaced with telephone call contacts during the lockdown, and parents were asked to give a subjective assessment about the child’s condition and describe ear-related complaints (ongoing AOM episodes, with/without tympanic membrane perforation, antibiotic treatments). The results of this survey were compared with the corresponding assessments reported 1 year prior. A statistically significant reduction in the mean number of AOM episodes and systemic antibiotic treatments during the COVID-19 first lockdown period compared with the previous year was reported, and 82% of parents had an impression of clinical improvement during the lockdown (Torretta et al., 2021).

Furthermore, a positive effect was reported on OME prevalence among Milanese children who attended an outpatient clinic during two pre-lockdown periods (May-June 2019 and January-February 2020) vs. the first post-lockdown period (May-June 2020) (Alde et al., 2021). It was found that OME prevalence dramatically decreased after the lockdown: 41%, 52%, and 2%, during the 1^st^, 2^nd^ and 3^rd^ periods, respectively. The resolution rate of OME was significantly higher in May-June 2020 when compared with the corresponding period of May-June 2019 (93% vs. 21%).

These findings were in line with results from a study conducted in *De Marchi*, Milan’s largest PED, aiming to measure the children’s flow, in terms of diagnosis related to any ear, nose, and throat (ENT) diseases during the lockdown (February-May 2020), compared with the corresponding previous period (February-May 2019) (Torretta et al., 2021). They found a substantial regional decrease in children’s attendance to that PED; this effect was particularly noticeable when the analysis was restricted to ENT diagnoses (80.4% vs. 19.5%, February-May 2019 and February-May 2020, respectively; *p*-value < 0.001), including middle ear infections (92.8% vs. 7.2%). In addition, non-complicated AOM episodes more frequently occurred in February-May 2019 (92.0% vs. 8.0%), but no significant differences were found between the number of patients with complicated middle ear diseases (95.8% vs. 4.2%).

**Table 2** summarizes current worldwide data on AOM burden reduction. A reduction in AOM burden was observed in many countries. In France, Angoulvant et al. (2021) conducted a time-series analysis for PED visits and related hospital admissions in the greater Paris area, from January 2017 to mid-April 2020. A global reduction in post-lockdown PED visits and hospital admissions was found (-68% and -45%, respectively), with a significant decrease (>70%) in the observed over expected rates of several infectious diseases, including AOM. These results are in line with the findings by Kuitunen et al. (2020), who evaluated the immediate effect of Finnish national lockdown on PED visits. A major decrease in the daily rate of PED visits was reported, along with an overall decrease in the number of hospitalized patients for any respiratory disease during the lockdown, compared with the previous period. A study from 27 PED across the USA has demonstrated that visit numbers decreased by 45.7% (range: 36.1%-96.9%) during the COVID-19 pandemic in 2020 when compared with the same period during 2017-2019. The largest decrease was for respiratory disorder visits (DeLaroche et al., 2021). A sharp decline was observed for both AOM and URTI: 75.1% and 69.9%, respectively.

**Table 2:** Reduction in otitis media burden during COVID-19 pandemic.

Country	Setting	COVID-19 Period	Comparative Period(s)	Variable	Rate Difference
Finland (Kuitunen, 2021)	Two hospitals	16/3/2020-12/4/2020	17/2/2020-15/3/2020	No. of AOM visits	-1%, -30%
USA (DeLaroche et al., 2021)	27 pediatric hospitals	15/3/2020-31/8/2020	15/3/2017-31/8/2017; 15/4/2018-31/8/2018;	Diseases of the ear and mastoid process	-68%
			15/4/2019-31/8/2019		
France (Angoulvant et al., 2021)	6 Paris area hospitals	18/3/2020-19/4/2020	1/1/2017-17/3/2020	No. AOM referrals	-70%
			(Time interrupted)		
Spain (Enrique et al., 2021)	Tertiary pediatric hospital	1/1/2020-30/6/2020	1/1/2010-31/12/2019	No. of mastoiditis cases	+45%
			(Time interrupted)		
Italy (Iannella et al., 2021)	Telephone/telemedicine contact with families of children with OM	1/2/2020-30/4/2020	1/2/2019-30/4/2019	No of AOM episodes,	-81%
				No. of otorrhea episodes,	-97%
				No. of ABx/month	-89%
USA (DeLaroche et al., 2021)	Tertiary and community health providers	1/1/2020-13/3/2020;	1/1/2019-13/3/2019;	No. of tympanostomy tube procedures	-64%
		1/6/2020-13/12/2020	1/6/2019-13/12/2019		
		(post-lockdown)			
Italy (Alde et al., 2021)	Tertiary pediatric hospital	1/5/2020-30/6/2020	1/5/2019-30/6/2019;	No. of children with OME,	-40%
		(post-lockdown)	1/1/2020-29/2/2020;	No. of children with type B tympanometry	-95%
The Netherlands (van de Pol et al., 2021)	Tertiary hospital and clinics network	1/3/2020-31/5/2020	1/3/2019-31/5/2019	No. of AOM episodes	-10%
USA (Ramgopal et al., 2021)	37 Children’s hospitals	1/1/20-31/12/20	1/1 to 31/12 for each year: 2010-2019	No. of AOM visits	-55%
Italy (Torretta et al., 2021)	Pediatric emergency Department	21/2/2020-4/5/2020	1/2/2019-21/2/2020	No. of AOM visits	-92%
Germany (Rohe et al., 2021)	146 ENT practice centers	Q2-Q3 2020	Q2-Q3 2019	No. of AOM visits	-43%
UK (Stansfield et al., 2021)	One large center	17/3/2020-17/6/2020	17/3/2019-17/3/2019	No. of emergency department OM visits	-86%
Italy (Torretta et al., 2020)	Telephone/telemedicine contact with families of children with OM	9/3/2020-19/5/2020	1/2/2020-30/4/2020	No. of AOM episodes	-90%
UK (Quraishi et al., 2021)	3 secondary care ENT departments	1/3/2020-28/2/2021	1/3/2019-29/2/2020	No. of AOM visits	-26.9%
				No. of acute mastoiditis cases	-14.3%
The Netherlands (Hullegie et al., 2021)	Julius General Practitioners’ Network	1/3/2020-28/2/2021	1/3/2019-29/2/2020	AOM, 0-2 years	-47.6%
				AOM, 2-6 years	-33.7%
				AOM, 6-12 years	-6.8%
Switzerland (Bucher et al., 2021)	Tertiary referral center	16/3/2020-26/4/2020	16/3/2019-26/4/2019	AOM, all ages	-79.5%

AOM, acute otitis media; MEF, middle ear fluid, ABx, antibiotics; OM, otitis media.

However, not all studies reported a downward trend in OM burden. A Spanish study showed that although there was no increase in the total ENT infections during January-April 2020 when compared with the same period in 2010-2019, there was a significant increase in complicated cases of mastoiditis (e.g., subperiosteal abscess, facial nerve paralysis, intracranial abscess). Although 54% of these patients were exposed to COVID-19 patients, only 15.4% had IgG antibodies (Enrique et al., 2021). The authors explained the increased complication rates might be explained due to lack of staff during the initial pandemic, and that public fear of getting infected may have prevented patients and parents from seeking medical treatment earlier.

Based on these reports, we speculate that vigorous lockdown measures also had a critical impact on the spread of many infectious diseases other than COVID-19 in children. Data are still accumulating.

## Reduction in Antibiotics Prescription Rates for AOM

Data regarding antibiotics prescription rates for AOM during the COVID-19 pandemic are limited. The COVID-19 era provided a unique opportunity for healthcare providers to utilize treatment guidelines for common childhood infections, such as AOM. The “Choosing Wisely” campaigns, presented in >20 countries, provide statements regarding when to use antibiotics, to avoid unnecessary treatment of viral infections (Leis et al., 2020). New toolkits, such as the “Cold Standard toolkit”, published by the Canadian College of Family Physicians, as part of the “Choosing Wisely” campaign, provide information on how to use antibiotics in virtual care visits during the COVID-19 era (https://choosingwiselycanada.org/perspective/the-cold-standard/). This campaign encourages a conversation between clinicians and patients to avoid antibiotic overuse.

In general, a delayed prescription is accepted for AOM cases; the prescription is filled and purchased only if symptoms persist (Spurling et al., 2017). As described by Leis et al. (2020), infants >6 months with otalgia should be seen in person only when symptoms persist >48h, fever >39°C despite antipyretic medications, or in ill-looking children. Otherwise, a virtual visit can take place, and the child can be treated with oral pain analgesics, according to the Canadian Pediatric Society guidelines on AOM management (Le Saux et al., 2016). However, virtual visits are limited in their diagnostic accuracy, and therefore may eventually lead to over-prescription of antibiotics (Leis et al., 2020).

A Scottish study examined the pandemic’s impact on outpatient antibiotics prescriptions rates, in comparison with the preceding year. It was shown that initially, there was a sharp increase in the numbers of prescriptions used for respiratory infections, followed by a decrease for all age groups, which was more pronounced in children aged 0-4 years. They suggested that the initial peak was due to “just-in-case” prescriptions. The decrease may be due to fewer URTI episodes and increased self-care practice and a reduced number of patients with bacterial infections presenting to their general practitioners (GPs) (Malcolm et al., 2020).

A Dutch study compared the number of GPs’ consultations and antibiotic prescription rates during March-May 2019, with the same period in 2020. The number of respiratory/ear infections decreased during the pandemic period from 16,672 to 15,580 and the antibiotics prescription rate decreased from 21% to 13%, respectively, without an increase in acute mastoiditis cases (van de Pol et al., 2021).

A web-based survey among 169 Israeli pediatricians circulated during the pandemic aimed to evaluate the frequency of telemedicine use, as well as its influence on decision-making in clinical scenarios, such as AOM. They reported an increase in daily use of text messages, pictures, and videoconference from 24%, 15%, and 1% before the pandemic to 40%, 40%, and 12% during the lockdown, respectively. Interestingly, there was a high likelihood of prescribing antibiotics for suspected AOM *via* telemedicine contacts (Grossman et al., 2020). 

## Otologic Surgery

### Changes in Concept

COVID-19 emergence required otologists to adopt a changed mindset for otologic office procedures and ear surgery. Otologic surgery, including drilling of the mastoid, is known to cause a substantial dispersion of small and large aerosols (Anschuetz et al., 2021; Chari et al., 2021; Hajiyev and Vilela, 2021; Merven and Loock, 2021; Sharma et al., 2021) and droplets (Sharma et al., 2020; Mohan et al., 2021; Sharma et al., 2021), and is a cause of concern regarding contamination in the operating room (OR). To prevent unnecessary risk of infection, otologic procedures were categorized as urgent versus elective, according to different authors and otolaryngological societies (Kozin et al., 2020; Leboulanger et al., 2020; Pattisapu et al., 2020; Saadi et al., 2020), suggesting that urgent operations should be performed, while all other operations should be postponed, depending on the pathology and the patient’s preference.

To overcome the obstacle of personal contamination through patient care, several methods have been studied/suggested (Cottrell et al., 2020; Ally et al., 2021; Chari et al., 2021): personal protective equipment (PPE) for clinics and the ORs, a shift to telehealth communication, environmental protection (microscope draping, tents), modification of surgical settings and alteration of surgical techniques (actively preferring endoscopic approaches, use of exoscopes, use of enhanced smoke/suction devices, povidone-iodine irrigation of mastoid). SARS-CoV-2 testing is pre-operatively advised, and if the test is negative, additional precautions may not be necessary. When SARS-CoV-2 testing is not possible, or when testing positive, extra care should be taken as listed below.

### Personal Protective Equipment

PPE can be divided into two categories: 1) respiratory protection (N95 respirator, powered air-purifying respirator [PAPR]), and 2) body protection, including eye protection, sterile and waterproof clothes around the neck, and disposable cap, gown, overshoes, and gloves. PPE is advised for any surgery performed, and especially for procedures with a high aerosol dispersion potential, such as mastoidectomy (Ayache and Schmerber, 2020; Gordon et al., 2020; Kozin et al., 2020; Leboulanger et al., 2020; Sharma et al., 2021).

### Surgical Setting

It was advised that the minimal number of staff enter the OR, and only the most skilled surgeon operating (Kozin et al., 2020). It is advised that for adults, VTI should be performed under local anesthesia. For pediatric cases, bag ventilation is not advised, and all procedures should be performed with endotracheal intubation. Operating rooms with negative pressure ventilation should be used as with a designed filtration system (Leboulanger et al., 2020). 

### Surgical Technique

Regarding surgical techniques, the endoscopic approach is preferred, if possible (Ayache et al., 2021). When a mastoidectomy is unavoidable, it should be performed without drilling, if possible, and by using instruments such as a hammer, gouge, and/or curette, together with a continuous high-powered suction that should be placed next to the surgical field. The use of monopolar cautery and laser may result in an increased risk of viral dissemination, and thus should be avoided (Ayache and Schmerber, 2020; Kozin et al., 2020; Lavinsky et al., 2020; Leboulanger et al., 2020; Saadi et al., 2020). Exoscopes should be chosen in place of microscopes, as exoscopes place the surgeon and staff a more secure distance from the surgical field (Gordon et al., 2020; Kozin et al., 2020; Ally et al., 2021; Ridge et al., 2021; Tu et al., 2021).

### Environmental Protection

Various methods of surgical field isolation for better staff protection have been proposed (Cottrell et al., 2020; Panda et al., 2020; Chari et al., 2021; Hajiyev and Vilela, 2021). **Table 3** describes the proposed materials. All these methods aim to isolate the surgical field to prevent aerosol and particle dispersion. Environmental protection uses commonly available surgical drapes and other equipment traditionally present in the OR. It is important to note that most of the proposed methods are prototypes (McCarty et al., 2021), and have not yet been rigorously proven.

**Table 3:** Draping techniques during otologic surgery.

Author	Drape “tent” (Other than Otological Surgical Drape/4K 3D Exoscope Drape)	Accessories	A place for Surgical Assistant and Instrument Table	Ease of Construction (Yes/No)	Cost	Name of Installation
Panda et al. (2020)	Steri-Drape (3M)	Gottingen laser support table	+	Y	+	
Cottrell et al. (2020)	C-arm draping	–	–	N	++	
(Hajiyev and Vilela, 2021)	Modified chair drape	–	–	Y	+	
Chari et al. (2021)	Steri-Drape (3M)	–	–	Y	+	Ototent 1
Chari et al. (2021)	Modified Zeiss OPMI microscope drape (Carl Zeiss, Meditec AG, Germany)	–	–	Y	++	Ototent 2
Tolisano et al. (2020)	Modified Zeiss OPMI microscope drape (Carl Zeiss, Meditec AG, Germany)	PVC pipes as a specialized frame,	–	N	++	**Covid-19 Airway Management Isolation Chamber with otologic modification (CAMIC-Ear)**
		Sterile bags				
Ally et al. (2021)	A plastic sterile drape (3M Steri-Drape 1015)	–	–	Y	+	
Lawrence et al. (2020)	*CE-marked* sterile polyethylene drape with a custom-made hole over the lens cap	‘L’ support	–	Y	+	

+, not expensive; ++, expensive.

### Changes in Ventilating Tube Surgery

Mohan et al. examined the risk of aerosol-generating procedures, such as suctioning of MEF during ventilating tube insertion (VTI), by performing a cadaveric simulation of bedside myringotomy and fluorescein-labeled fluid injection into the middle ear to examine the potential risk (Mohan et al., 2021). Image analysis showed no fluid in the proximal external auditory canal nor the ear speculum following the procedure and suctioning. Unlike first speculations, there was no measured increase in aerosol particle numbers during VTI (Campiti et al., 2021).

Another report from Milan addressed the clinical activities pertinent to pediatric OM and modifications of surgical waiting lists during the COVID-19 pandemic, with patient selection based upon the priority of certain conditions, as defined by the Italian Society of Otorhinolaryngology-Head and Neck Surgery (Torretta et al., 2020). Priority for VTI surgery was granted to candidates with persistent OME, causing a negative impact on language development. During the pandemic, VTI rates significantly decreased among Bostonian children: the age of patients undergoing surgery increased, and more children were sent for surgery in a tertiary setting (Diercks and Cohen, 2021). It was also reported that in young Floridian children, the prevalence of intraoperative OME during the COVID-19 pandemic was significantly lower compared with pre-COVID-19 as assessed during VTI surgery (65% vs. 83%, p <0.001) (Nguyen et al., 2021). 

## Telemedicine

Telemedicine is the branch of telehealth that connects patients-to-providers and providers-to-providers, for the delivery of healthcare at a distance. Although this development offered greater healthcare access, telemedicine is limited in accuracy and general acceptance by a lack of physical examination and real-time intervention, remaining as a service for special circumstances, or as an adjunct to in-office and in-hospital visits. Telemedicine has been well-received as a modality for patient visits. Caregivers and care providers alike report high satisfaction in both the convenience and the care provided by the service (McIntosh et al., 2014).

The COVID-19 pandemic significantly expanded the usage of telemedicine. The call for strict socially distancing, and patients’ fears of any medical environment, meant that any means for assessment was preferable to the risk of contracting the virus. The result has been more widespread use of newly developing technologies, permitting triage and assessment of patients remotely. For OM, evaluation of the tympanic membrane and the middle ear by a tele-provider is conducted in one of two ways: video microscopy or video otoscopy, the latter of which also often permits pneumatic otoscopy. The exam is carried out by the patient’s family or a telemedicine facilitator, and findings may be transmitted to a provider in real-time, or recorded and delivered for interval review (Metcalfe et al., 2021).

### Types of Telemedicine

1. Home-based telemedicine (HBT) starts with a smartphone. Instrumentation in the home setting may be owned by the patient or may be sent to the family, in anticipation of a telemedicine visit. A hybrid model for telemedicine care, blending telemedicine with an in-person office visit, can be offered when further needs arise for better information or from the opportunity for a different interaction with the provider.
2. Facility-based telemedicine (FBT) requires that the patient be brought to a clinical setting with visualization technology available for use. Information is sent to a physician or advanced-practice provider located at a distance.

Two temporal modes of telemedicine exist:

1. *Synchronous telemedicine* involves real-time telephone or audiovisual interaction *via* smartphone, tablet, or computer with patients and families. This may occur in the patient’s own home in a direct-to-consumer model or an external facility.
2. *Asynchronous telemedicine* includes “store and forward” technology in which messages, images, and data are securely sent and subsequently reviewed and responded to within a certain time interval. The utilization of patient portals is often utilized.

### Telemedicine for AOM

**Figure 1:**
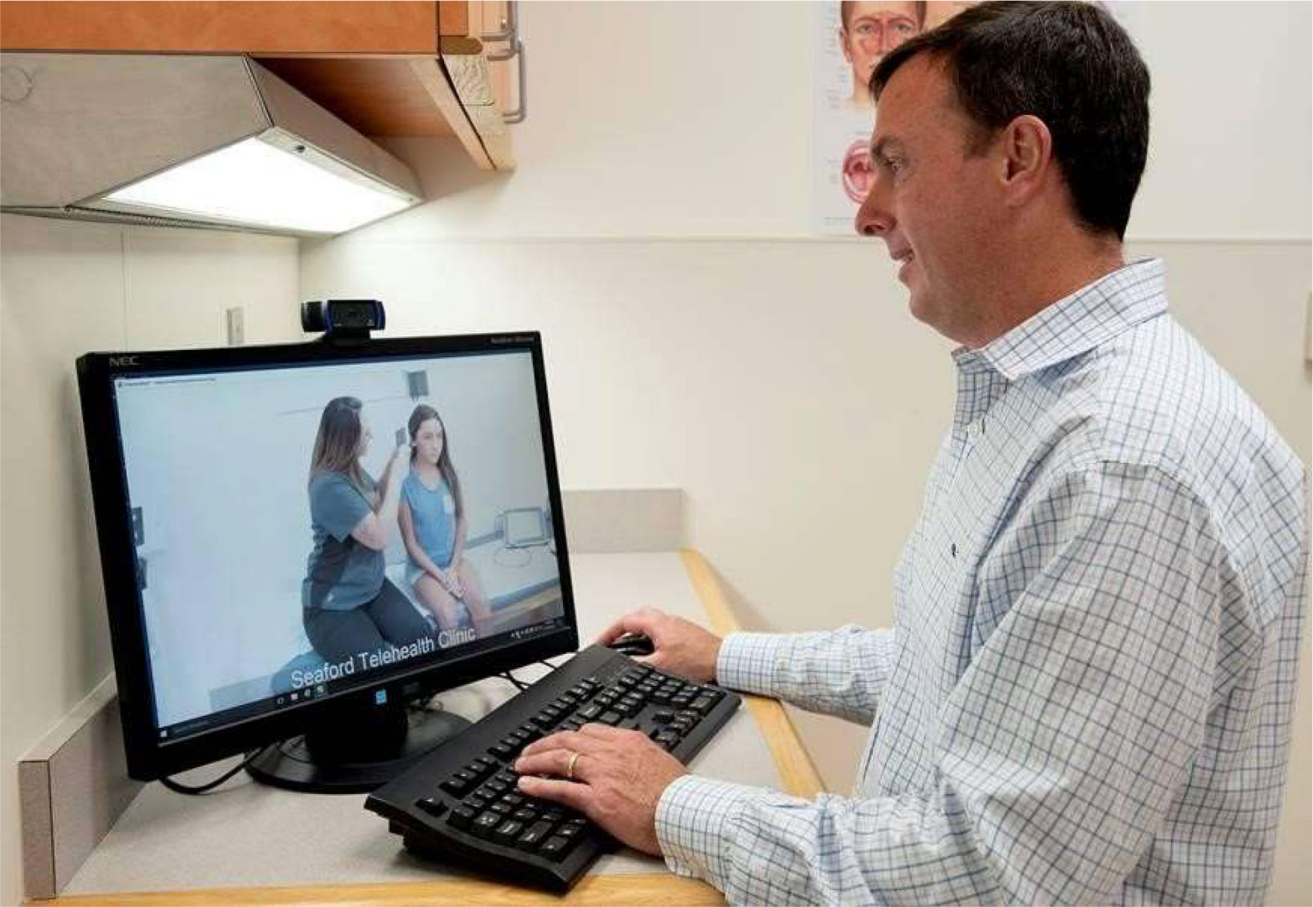
Medical assistant telemedicine in use. A physician provider to the far right is watching the medical assistant at the far left on the screen using a device to examine the ear of a patient. The device will relay images of the patient’s ear exam to the physician (Photo used with permission of A. Saporito. Image courtesy of Dr. Patrick Barth).

**Figure 2:**
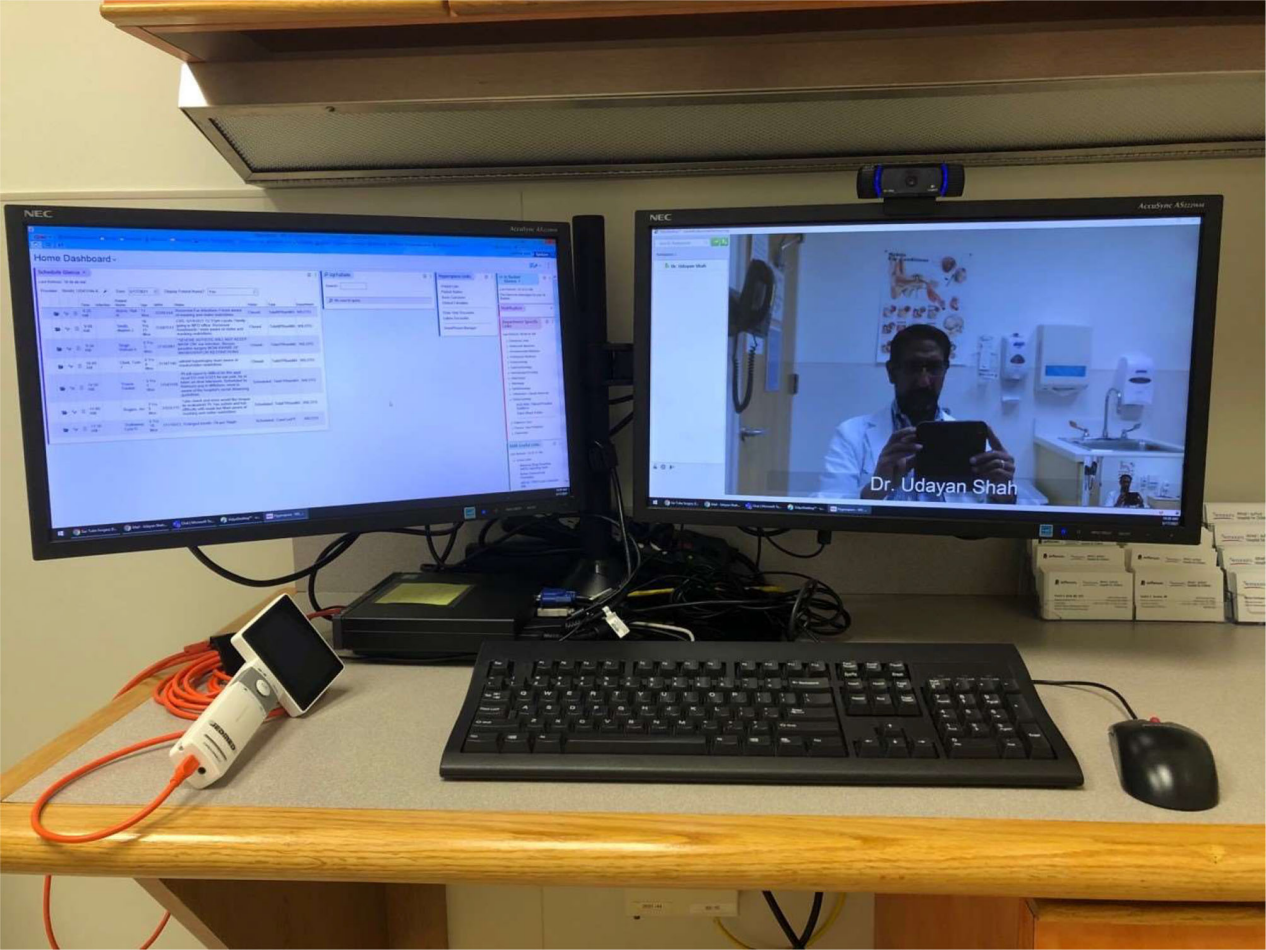
Possible telemedicine setup. A two-screen setup allows the medical record to be seen on the left, while the examination can be seen on the right. A white hand-held video-otoscope is seen to the left of the keyboard (Image courtesy of Dr. Udayan Shah).

**Figure 3:**
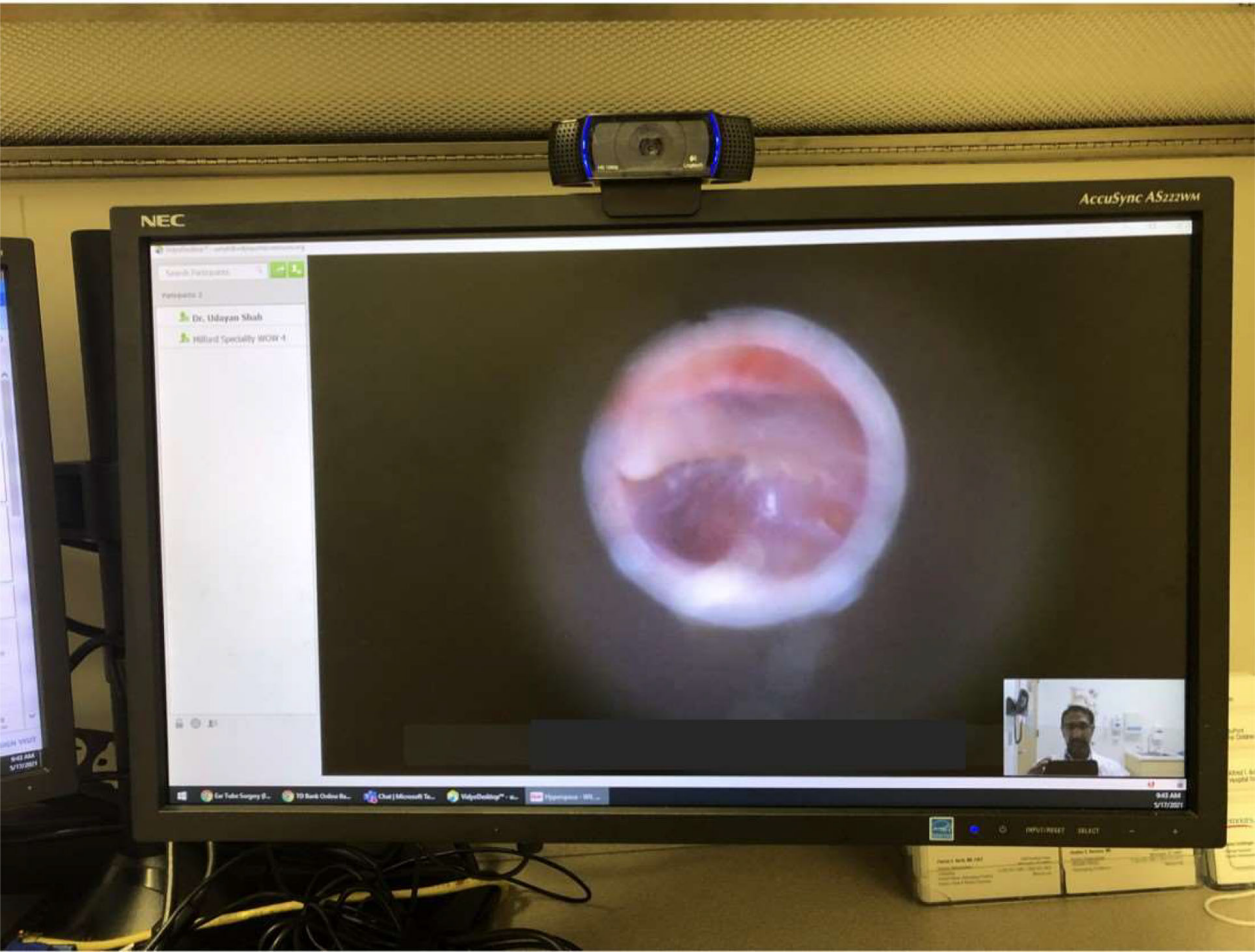
Otoscopy *via* telemedicine. The camera is at top of the monitor.

Because AOM is the most common reason for pediatric outpatient visits, HBT can offer earlier, more accurate diagnoses for primary care providers, meaning more judicious antibiotic usage, and better anticipation of impending complications. Telemedicine in AOM is equivalent to office-based visits regarding recommendations for surgery, additional testing, or routine follow-up (Kolb et al., 2021). The challenge of patient assessment in the setting of potential OM is in direct visualization of middle ear structures. Another important factor for this process is the attainment of satisfactory image quality. Regulatory restrictions are an additional limitation at present. Licensure, and credentialing providers, limit the extent of care that can be provided. In some organizations, the provider is a trained medical assistant. Because any healthcare provider can be trained, this approach to a hybrid model of telemedicine and in-person care is termed “Provider Assisted Telemedicine” (PAT). Additionally, tympanometry can be performed when performing FBT for AOM: the delivering provider obtains a tympanogram which is then messaged to the remote, directing provider (**Figures 1**–**3**).

Interestingly, studies on telemedicine and antibiotic prescription before the COVID-19 era have shown that children seen by telemedicine visits received less diagnostic testing and would not seek antibiotic treatment if they have received a clear explanation on diagnosis, safety, treatment plan, and reassurance (Mangione-Smith et al., 1999).

Telemedicine was found to help guide decision-making regarding medical and surgical management for AOM, though a consensus in the literature on the benefits of telemedicine in pediatrics for triage and assessment of otitis media has not yet been established. Telemedicine continues today to demonstrate its utility during the COVID-19 pandemic.

## Conclusion

The paucity of reports of living COVID-19 patients with AOM, particularly for children, can be explained by the variability in clinical severity and viral load (with more severe clinical course portending a higher viral titer), very low incidence of myringotomies to aspirate MEF, low levels of passive reflux from the nasopharynx, a low predilection for SARS-CoV-2 to invade the middle ear mucosa and inadequate detection technique. In addition, hospital admission reports and insurance-claims data showed a substantial decrease in OM visit rates and even related complications during the COVID-19 pandemic periods, compared with previous years. Otologic surgeons identified several methods to minimize the spread of the COVID-19 virus. These methods include PPE, environmental protection, and alteration of surgical methods and techniques. The combination of these methods may best protect the surgeon and the staff against this ever-changing disease.

Future research should focus on OM burden following the introduction of SARS-2-CoV vaccines (currently approved only for children >12 years), relaxation of lockdown measures, and re-emergence of viral URTIs other than SARS-CoV-2, and appearance of new SARS-CoV-2 variants.

## Author Contributions

TM, JP, and STa contributed to conception and design of the study. TM and STa identified and assigned areas of review. TM, JP, and STa wrote the first draft of the manuscript. US, STa, STo, PM, AK, and PB wrote sections of the manuscript. All authors contributed to manuscript revision, read, and approved the submitted version.

## Conflict of Interest

The authors declare that the research was conducted in the absence of any commercial or financial relationships that could be construed as a potential conflict of interest.

## Publisher’s Note

All claims expressed in this article are solely those of the authors and do not necessarily represent those of their affiliated organizations, or those of the publisher, the editors and the reviewers. Any product that may be evaluated in this article, or claim that may be made by its manufacturer, is not guaranteed or endorsed by the publisher.

## References

[B1] Alde M. Di Berardino F. Marchisio P. Cantarella G. Ambrosetti U. Consonni D.. (2021). Effects of COVID-19 Lockdown on Otitis Media With Effusion in Children: Future Therapeutic Implications. Otolaryngol. Head Neck Surg. 165 (5), 710–715. doi: 10.1177/0194599820987458 33494659 PMC7841252

[B2] Ally M. Kullar P. Mochloulis G. Vijendren A. (2021). Using a 4K Three-Dimensional Exoscope System (Vitom 3D) for Mastoid Surgery During the Coronavirus Disease 2019 Pandemic. J. Laryngol. Otol. 135 (3), 273–275. doi: 10.1017/S002221512100044X 33517922 PMC7925976

[B3] Angoulvant F. Ouldali N. Yang D. D. Filser M. Gajdos V. Rybak A.. (2021). Coronavirus Disease 2019 Pandemic: Impact Caused by School Closure and National Lockdown on Pediatric Visits and Admissions for Viral and Nonviral Infections-A Time Series Analysis. Clin. Infect. Dis. 72 (2), 319–322. doi: 10.1093/cid/ciaa710 33501967 PMC7314162

[B4] Anschuetz L. Yacoub A. Buetzer T. Fernandez I. J. Wimmer W. Caversaccio M. (2021). Quantification and Comparison of Droplet Formation During Endoscopic and Microscopic Ear Surgery: A Cadaveric Model. Otolaryngol. Head Neck Surg. 164 (6), 1208–1213. doi: 10.1177/0194599820970506 33138717 PMC7642824

[B5] Ayache S. Kutz W. Isaacson B. Badr-El-Dine M. Nogueira J. F. Marchioni D.. (2021). COVID-19 and Ear Endoscopy in Otologic Practices. Eur. Arch. Otorhinolaryngol. 278 (6), 2133–2135. doi: 10.1007/s00405-020-06309-9 32876726 PMC7466917

[B6] Ayache S. Schmerber S. (2020). Covid-19 and Otologic/Neurotologic Practices: Suggestions to Improve the Safety of Surgery and Consultations. Otol. Neurotol. 41 (9), 1175–1181. doi: 10.1097/MAO.0000000000002851 32925833

[B7] Bucher S. Neumann A. S. Meerwein C. M. Holzmann D. Soyka M. B. (2021). Reduction of Otorhinolaryngological Consultations Due to the COVID-19 Lockdown and Its Impact on Disease Progression. Swiss. Med. Wkly. 151, w30068. doi: 10.4414/smw.2021.w30068 34668689

[B8] Bulut Y. Guven M. Otlu B. Yenisehirli G. Aladag I. Eyibilen A.. (2007). Acute Otitis Media and Respiratory Viruses. Eur. J. Pediatr. 166 (3), 223–228. doi: 10.1007/s00431-006-0233-x 16967296 PMC7086696

[B9] Campiti V. J. Ye M. J. Sharma D. Matt B. H. Mitchell R. M. Ting J. Y.. (2021). Aerosol Generation During Myringotomy With Tympanostomy Tube Insertion: Implications for Otolaryngology in the COVID-19 Era. Otolaryngol. Head Neck Surg. 165 (4), 532–535. doi: 10.1177/0194599821989626 33557705

[B10] Chari D. A. Workman A. D. Chen J. X. Jung D. H. Abdul-Aziz D. Kozin E. D.. (2021). Aerosol Dispersion During Mastoidectomy and Custom Mitigation Strategies for Otologic Surgery in the COVID-19 Era. Otolaryngol. Head Neck Surg. 164 (1), 67–73. doi: 10.1177/0194599820941835 32660367 PMC7361126

[B11] Chong S. L. Soo J. S. L. Allen J. C. Jr. Ganapathy S. Lee K. P. Tyebally A.. (2020). Impact of COVID-19 on Pediatric Emergencies and Hospitalizations in Singapore. BMC Pediatr. 20 (1), 562. doi: 10.1186/s12887-020-02469-z 33353540 PMC7755581

[B12] Chonmaitree T. Alvarez-Fernandez P. Jennings K. Trujillo R. Marom T. Loeffelholz M. J.. (2015). Symptomatic and Asymptomatic Respiratory Viral Infections in the First Year of Life: Association With Acute Otitis Media Development. Clin. Infect. Dis. 60 (1), 1–9. doi: 10.1093/cid/ciu714 25205769 PMC4318943

[B13] Chonmaitree T. Revai K. Grady J. J. Clos A. Patel J. A. Nair S.. (2008). Viral Upper Respiratory Tract Infection and Otitis Media Complication in Young Children. Clin. Infect. Dis. 46 (6), 815–823. doi: 10.1086/528685 18279042 PMC2744371

[B14] Cottrell J. Lui J. Le T. Chen J. (2020). An Operative Barrier System for Skull Base and Mastoid Surgery: Creating a Safe Operative Theatre in the Era of COVID-19. J. Otolaryngol. Head Neck Surg. 49 (1), 71. doi: 10.1186/s40463-020-00471-0 33023663 PMC7537966

[B15] DeLaroche A. M. Rodean J. Aronson P. L. Fleegler E. W. Florin T. A. Goyal M.. (2021). Pediatric Emergency Department Visits at US Children’s Hospitals During the COVID-19 Pandemic. Pediatrics 147 (4), e2020039628. doi: 10.1542/peds.2020-039628 33361360

[B16] Diercks G. R. Cohen M. S. (2021). The Effect of the COVID-19 Pandemic on Pediatric Tympanostomy Tube Placement. Otolaryngol. Head Neck Surg. 1945998211008916. doi: 10.1177/01945998211008916 33940984

[B17] Drosten C. Gunther S. Preiser W. van der Werf S. Brodt H. R. Becker S.. (2003). Identification of a Novel Coronavirus in Patients With Severe Acute Respiratory Syndrome. N. Engl. J. Med. 348 (20), 1967–1976. doi: 10.1056/NEJMoa030747 12690091

[B18] Enrique G. L. Margarita B. B. Angel M. J. Saturnino S. S. Maria Jesus D. R. (2021). COVID-19 and Severe ENT Infections in Pediatric Patients. IS There a Relationship? Int. J. Pediatr. Otorhinolaryngol. 145, 110714. doi: 10.1016/j.ijporl.2021.110714 33894522 PMC8051009

[B19] Ferrero F. Ossorio M. F. Torres F. A. Debaisi G. (2021). Impact of the COVID-19 Pandemic in the Paediatric Emergency Department Attendances in Argentina. Arch. Dis. Child 106 (2), e5. doi: 10.1136/archdischild-2020-319833 32554509

[B20] Fidan V. (2020). New Type of Corona Virus Induced Acute Otitis Media in Adult. Am. J. Otolaryngol. 41 (3), 102487. doi: 10.1016/j.amjoto.2020.102487 32336572 PMC7161479

[B21] Finkelstein Y. Maguire B. Zemek R. Osmanlliu E. Kam A. J. Dixon A.. (2021). Effect of the COVID-19 Pandemic on Patient Volumes, Acuity, and Outcomes in Pediatric Emergency Departments: A Nationwide Study. Pediatr. Emerg. Care. 37 (8), 427–434. doi: 10.1097/PEC.0000000000002484 34074990 PMC8327936

[B22] Frazier K. M. Hooper J. E. Mostafa H. H. Stewart C. M. (2020). SARS-CoV-2 Virus Isolated From the Mastoid and Middle Ear: Implications for COVID-19 Precautions During Ear Surgery. JAMA Otolaryngol. Head Neck Surg. 146 (10), 964–966. doi: 10.1001/jamaoto.2020.1922 32701126 PMC7378866

[B23] Gordon S. A. Deep N. L. Jethanamest D. (2020). Exoscope and Personal Protective Equipment Use for Otologic Surgery in the Era of COVID-19. Otolaryngol. Head Neck Surg. 163 (1), 179–181. doi: 10.1177/0194599820928975 32423361

[B24] Grossman Z. Chodick G. Reingold S. M. Chapnick G. Ashkenazi S. (2020). The Future of Telemedicine Visits After COVID-19: Perceptions of Primary Care Pediatricians. Isr. J. Health Policy Res. 9 (1), 53. doi: 10.1186/s13584-020-00414-0 33081834 PMC7573530

[B25] Hajiyev Y. Vilela R. J. (2021). Cost-Effective Microscopic Draping: COVID-19 Umbrella. Ear. Nose. Throat. J. 1455613211005113. doi: 10.1177/01455613211005113 33765854

[B26] Hullegie S. Schilder A. G. M. Marchisio P. de Sevaux J. L. H. van der Velden A. W. van de Pol A. C.. (2021). A Strong Decline in the Incidence of Childhood Otitis Media During the COVID-19 Pandemic in the Netherlands. Front. Cell Infect. Microbiol. 11, 768377. doi: 10.3389/fcimb.2021.768377 34790591 PMC8591181

[B27] Iannella G. Magliulo G. Lechien J. R. Maniaci A. Perrone T. Frasconi P. C.. (2021). Impact of COVID-19 Pandemic on the Incidence of Otitis Media With Effusion in Adults and Children: A Multicenter Study. Eur. Arch. Otorhinolaryngol. 1–7. doi: 10.1007/s00405-021-06958-4 PMC8255053 34218309

[B28] Jee Y. (2020). WHO International Health Regulations Emergency Committee for the COVID-19 Outbreak. Epidemiol. Health 42, e2020013. doi: 10.4178/epih.e2020013 32192278 PMC7285442

[B29] Kolb C. M. Born K. Banker K. Barth P. Aaronson N. L. (2021). Comparing Telehealth With Office-Based Visits for Common Pediatric Otolaryngology Complaints. Int. J. Pediatr. Otorhinolaryngol. 145, 110712. doi: 10.1016/j.ijporl.2021.110712 33887549

[B30] Kozin E. D. Remenschneider A. K. Blevins N. H. Jan T. A. Quesnel A. M. Chari D. A.. (2020). American Neurotology Society, American Otological Society, and American Academy of Otolaryngology - Head and Neck Foundation Guide to Enhance Otologic and Neurotologic Care During the COVID-19 Pandemic. Otol. Neurotol. 41 (9), 1163–1174. doi: 10.1097/MAO.0000000000002868 32925832

[B31] Kuitunen I. (2021). Social Restrictions Due to COVID-19 and the Incidence of Intoxicated Patients in Pediatric Emergency Department. Ir. J. Med. Sci. 1–3. doi: 10.1007/s11845-021-02686-0 PMC8213037 34145548

[B32] Kuitunen I. Artama M. Makela L. Backman K. Heiskanen-Kosma T. Renko M. (2020). Effect of Social Distancing Due to the COVID-19 Pandemic on the Incidence of Viral Respiratory Tract Infections in Children in Finland During Early 2020. Pediatr. Infect. Dis. J. 39 (12), e423–e4e7. doi: 10.1097/INF.0000000000002845 32773660

[B33] Lavinsky J. Kosugi E. M. Baptistella E. Roithmann R. Dolci E. Ribeiro T. K.. (2020). An Update on COVID-19 for the Otorhinolaryngologist - a Brazilian Association of Otolaryngology and Cervicofacial Surgery (ABORL-CCF) Position Statement. Braz. J. Otorhinolaryngol. 86 (3), 273–280. doi: 10.1016/j.bjorl.2020.04.002 32371055 PMC7151294

[B34] Lawrence R. J. O’Donoghue G. Kitterick P. O’Donoghue K. Hague R. Mitchell L.. (2020). Recommended Personal Protective Equipment for Cochlear Implant and Other Mastoid Surgery During the COVID-19 Era. Laryngoscope 130 (11), 2693–2699. doi: 10.1002/lary.29014 32720316

[B35] Leboulanger N. Sagardoy T. Akkari M. Ayari-Khalfallah S. Celerier C. Fayoux P.. (2020). COVID-19 and ENT Pediatric Otolaryngology During the COVID-19 Pandemic. Guidelines of the French Association of Pediatric Otorhinolaryngology (AFOP) and French Society of Otorhinolaryngology (SFORL). Eur. Ann. Otorhinolaryngol. Head Neck Dis. 137 (3), 177–181. doi: 10.1016/j.anorl.2020.04.010 32312676 PMC7165275

[B36] Leis J. A. Born K. B. Theriault G. Ostrow O. Grill A. Johnston K. B. (2020). Using Antibiotics Wisely for Respiratory Tract Infection in the Era of Covid-19. BMJ 371, m4125. doi: 10.1136/bmj.m4125 33187951

[B37] Le Saux N. Robinson J. L. Canadian Paediatric Society Infectious Diseases and Immunization Committee (2016). Management of Acute Otitis Media in Children Six Months of Age and Older. Paediatr. Child Health 21 (1), 39–50. doi: 10.1093/pch/21.1.39 26941560 PMC4758427

[B38] Liaw J. Saadi R. Patel V. A. Isildak H. (2021). Middle Ear Viral Load Considerations in the COVID-19 Era: A Systematic Review. Otol. Neurotol. 42 (2), 217–226. doi: 10.1097/MAO.0000000000002986 33201081 PMC7803391

[B39] Lin C. F. Huang Y. H. Cheng C. Y. Wu K. H. Tang K. S. Chiu I. M. (2020). Public Health Interventions for the COVID-19 Pandemic Reduce Respiratory Tract Infection-Related Visits at Pediatric Emergency Departments in Taiwan. Front. Public Health 8, 604089. doi: 10.3389/fpubh.2020.604089 33392141 PMC7772199

[B40] Malcolm W. Seaton R. A. Haddock G. Baxter L. Thirlwell S. Russell P.. (2020). Impact of the COVID-19 Pandemic on Community Antibiotic Prescribing in Scotland. JAC. Antimicrob. Resist. 2 (4), dlaa105. doi: 10.1093/jacamr/dlaa105 34192254 PMC7798936

[B41] Mangione-Smith R. McGlynn E. A. Elliott M. N. Krogstad P. Brook R. H. (1999). The Relationship Between Perceived Parental Expectations and Pediatrician Antimicrobial Prescribing Behavior. Pediatrics 103 (4 Pt 1), 711–718. doi: 10.1542/peds.103.4.711 10103291

[B42] Matusiak M. Schurch C. M. (2020). Expression of SARS-CoV-2 Entry Receptors in the Respiratory Tract of Healthy Individuals, Smokers and Asthmatics. Respir. Res. 21 (1), 252. doi: 10.1186/s12931-020-01521-x 32993656 PMC7523260

[B43] McBride J. A. Eickhoff J. Wald E. R. (2020). Impact of COVID-19 Quarantine and School Cancelation on Other Common Infectious Diseases. Pediatr. Infect. Dis. J. 39 (12), e449–ee52. doi: 10.1097/INF.0000000000002883 33031142

[B44] McCarty E. B. Soldatova L. Brant J. A. Newman J. G. (2021). Innovations in Otorhinolaryngology in the Age of COVID-19: A Systematic Literature Review. World J. Otorhinolaryngol. Head Neck Surg. doi: 10.1016/j.wjorl.2021.01.001 PMC7825952 33520334

[B45] McIntosh S. Cirillo D. Wood N. Dozier A. M. Alarie C. McConnochie K. M. (2014). Patient Evaluation of an Acute Care Pediatric Telemedicine Service in Urban Neighborhoods. Telemed. J. E. Health 20 (12), 1121–1126. doi: 10.1089/tmj.2014.0032 25290233 PMC4270158

[B46] McMillan P. Dexhiemer T. Neubig R. R. Uhal B. D. (2021). COVID-19-A Theory of Autoimmunity Against ACE-2 Explained. Front. Immunol. 12, 582166. doi: 10.3389/fimmu.2021.582166 33833750 PMC8021777

[B47] Merven M. Loock J. W. (2021). The Article “Demonstration and Mitigation of Aerosol and Particle Dispersion During Mastoidectomy Relevant to the Covid-19 Era” by Chen Jx, Et al. [Epub Ahead of Print] Refers. Otol. Neurotol. 42 (2), 346–347. doi: 10.1097/MAO.0000000000002905 33122505

[B48] Metcalfe C. Muzaffar J. Orr L. Coulson C. (2021). A Systematic Review of Remote Otological Assessment Using Video-Otoscopy Over the Past 10 Years: Reliability and Applications. Eur. Arch. Otorhinolaryngol. 278 (12), 4733–4741. doi: 10.1007/s00405-020-06596-2 33486567 PMC7828099

[B49] Mohan S. Workman A. Barshak M. Welling D. B. Abdul-Aziz D. (2021). Considerations in Management of Acute Otitis Media in the COVID-19 Era. Ann. Otol. Rhinol. Laryngol. 130 (5), 520–527. doi: 10.1177/0003489420958443 32911957

[B50] Nguyen D. K. Jueng J. Maul T. M. Wei J. L. (2021). Middle Ear Effusion Prevalence at Time of Tympanostomy Before and During COVID-19 Pandemic. Int. J. Pediatr. Otorhinolaryngol. 147, 110785. doi: 10.1016/j.ijporl.2021.110785 34116322

[B51] Nokso-Koivisto J. Pitkaranta A. Blomqvist S. Kilpi T. Hovi T. (2000). Respiratory Coronavirus Infections in Children Younger Than Two Years of Age. Pediatr. Infect. Dis. J. 19 (2), 164–166. doi: 10.1097/00006454-200002000-00016 10694007

[B52] Panda N. K. Agarwal G. Hage N. Balaji R. (2020). A Novel Technique of Draping for Otologic Surgery: Mitigation of Aerosol Generation During Covid Pandemic. Indian J. Otolaryngol. Head Neck Surg. 72 (4), 1–3. doi: 10.1007/s12070-020-01983-x 32837940 PMC7374067

[B53] Pattisapu P. Evans S. S. Noble A. R. Norton S. J. Ou H. C. Sie K. C. Y.. (2020). Defining Essential Services for Deaf and Hard of Hearing Children During the COVID-19 Pandemic. Otolaryngol. Head Neck Surg. 163 (1), 91–93. doi: 10.1177/0194599820925058 32366178

[B54] Pepper M. P. Leva E. Trivedy P. Luckey J. Baker M. D. (2021). Analysis of Pediatric Emergency Department Patient Volume Trends During the COVID-19 Pandemic. Med. (Baltimore). 100 (27), e26583. doi: 10.1097/MD.0000000000026583 PMC8270607 34232205

[B55] Pitkaranta A. Virolainen A. Jero J. Arruda E. Hayden F. G. (1998). Detection of Rhinovirus, Respiratory Syncytial Virus, and Coronavirus Infections in Acute Otitis Media by Reverse Transcriptase Polymerase Chain Reaction. Pediatrics 102 (2 Pt 1), 291–295. doi: 10.1542/peds.102.2.291 9685428

[B56] Quraishi N. Ray M. Srivastava R. Ray J. Quraishi M. S. (2021). A Multicentre Retrospective Cohort Study on COVID-19-Related Physical Interventions and Adult Hospital Admissions for ENT Infections. Eur. Arch. Otorhinolaryngol. 1–8. doi: 10.1007/s00405-021-07180-y PMC8607061 34807284

[B57] Raad N. Ghorbani J. Mikaniki N. Haseli S. Karimi-Galougahi M. (2021). Otitis Media in Coronavirus Disease 2019: A Case Series. J. Laryngol. Otol. 135 (1), 10–13. doi: 10.1017/S0022215120002741 33407978 PMC7804088

[B58] Ramgopal S. Pelletier J. H. Rakkar J. Horvat C. M. (2021). Forecast Modeling to Identify Changes in Pediatric Emergency Department Utilization During the COVID-19 Pandemic. Am. J. Emerg. Med. 49, 142–147. doi: 10.1016/j.ajem.2021.05.047 34111834 PMC8555971

[B59] Rasmussen S. A. Watson A. K. Swerdlow D. L. (2016). Middle East Respiratory Syndrome (MERS). Microbiol. Spectr. 4 (3). doi: 10.1128/microbiolspec.EI10-0020-2016 27337460

[B60] Ridge S. E. Shetty K. R. Lee D. J. (2021). Heads-Up Surgery: Endoscopes and Exoscopes for Otology and Neurotology in the Era of the COVID-19 Pandemic. Otolaryngol. Clin. North Am. 54 (1), 11–23. doi: 10.1016/j.otc.2020.09.024 33243372 PMC7522672

[B61] Rohe A. M. Kostev K. Sesterhenn A. M. (2021). [Impact of the COVID-19 Pandemic on Consultations and Diagnosis in ENT Practices in Germany]. Laryngorhinootologie. doi: 10.1055/a-1510-9686 34130328

[B62] Ruohola A. Meurman O. Nikkari S. Skottman T. Salmi A. Waris M.. (2006). Microbiology of Acute Otitis Media in Children With Tympanostomy Tubes: Prevalences of Bacteria and Viruses. Clin. Infect. Dis. 43 (11), 1417–1422. doi: 10.1086/509332 17083014 PMC7107988

[B63] Saadi R. A. Bann D. V. Patel V. A. Goldenberg D. May J. Isildak H. (2020). A Commentary on Safety Precautions for Otologic Surgery During the COVID-19 Pandemic. Otolaryngol. Head Neck Surg. 162 (6), 797–799. doi: 10.1177/0194599820919741 32286916

[B64] Sarateanu D. E. Ehrengut W. (1980). A Two Year Serological Surveillance of Coronavirus Infections in Hamburg. Infection 8 (2), 70–72. doi: 10.1007/BF01639150. 6248465 PMC7100827

[B65] Schmidt O. W. Allan I. D. Cooney M. K. Foy H. M. Fox J. P. (1986). Rises in Titers of Antibody to Human Coronaviruses OC43 and 229E in Seattle Families During 1975-1979. Am. J. Epidemiol. 123 (5), 862–868. doi: 10.1093/oxfordjournals.aje.a114315 3008551 PMC7110132

[B66] Sharma D. Campiti V. J. Ye M. J. Saltagi M. Carroll A. E. Ting J. Y.. (2021). Aerosol Generation During Cadaveric Simulation of Otologic Surgery and Live Cochlear Implantation. Laryngoscope. Investig. Otolaryngol. 6 (1), 129–136. doi: 10.1002/lio2.506 PMC7883621 33614941

[B67] Sharma D. Rubel K. E. Ye M. J. Campiti V. J. Carroll A. E. Ting J. Y.. (2020). Cadaveric Simulation of Otologic Procedures: An Analysis of Droplet Splatter Patterns During the COVID-19 Pandemic. Otolaryngol. Head Neck Surg. 163 (2), 320–324. doi: 10.1177/0194599820930245 32423287 PMC7240315

[B68] Spurling G. K. Del Mar C. B. Dooley L. Foxlee R. Farley R. (2017). Delayed Antibiotic Prescriptions for Respiratory Infections. Cochrane Database Syst. Rev. 9, CD004417. doi: 10.1002/14651858.CD004417.pub5. 28881007 PMC6372405

[B69] Stansfield J. Dobbs S. Harrison R. Lee K. Sharma S. Okour K.. (2021). Management of ENT Emergencies During the Coronavirus Disease 2019 Pandemic. J. Laryngol. Otol. 135 (2), 117–124. doi: 10.1017/S0022215121000530 33612142 PMC7900667

[B70] Stol K. Diavatopoulos D. A. Graamans K. Engel J. A. Melchers W. J. Savelkoul H. F.. (2012). Inflammation in the Middle Ear of Children With Recurrent or Chronic Otitis Media Is Associated With Bacterial Load. Pediatr. Infect. Dis. J. 31 (11), 1128–1134. doi: 10.1097/INF.0b013e3182611d6b 22668804

[B71] Tolisano A. M. Blood T. C. Jr. Riley C. A. Ruhl D. S. Hong S. S. (2020). The COVID-19 Airway Management Isolation Chamber (CAMIC) for Ears. Laryngoscope 130 (11), 2690–2692. doi: 10.1002/lary.28942 32602111 PMC7361893

[B72] Torretta S. Cantoni B. Bertolozzi G. Capaccio P. Milani G. P. Pignataro L.. (2021). Has Otitis Media Disappeared During COVID-19 Pandemic? A Fortuitus Effect of Domestic Confinement. J. Clin. Med. 10 (13), 2851. doi: 10.3390/jcm10132851 34199138 PMC8267642

[B73] Torretta S. Capaccio P. Coro I. Bosis S. Pace M. E. Bosi P.. (2021). Incidental Lowering of Otitis-Media Complaints in Otitis-Prone Children During COVID-19 Pandemic: Not All Evil Comes to Hurt. Eur. J. Pediatr. 180 (2), 649–652. doi: 10.1007/s00431-020-03747-9 32691131 PMC7370867

[B74] Torretta S. Capaccio P. Gaffuri M. Gaini L. M. Borin M. Maruca A.. (2020). ENT Management of Children With Adenotonsillar Disease During COVID-19 Pandemic. Ready to Start Again? Int. J. Pediatr. Otorhinolaryngol. 138, 110145. doi: 10.1016/j.ijporl.2020.110145 32499073 PMC7253984

[B75] Tu N. Bojrab D. 2nd Sioshansi P. Lin K. Hong R. Bojrab D.. (2021). Exoscope-Assisted Otologic Surgery During the COVID-19 Pandemic. Otol. Neurotol. 42 (3), e378–e3e9. doi: 10.1097/MAO.0000000000002916 33122504

[B76] Vabret A. Mourez T. Dina J. van der Hoek L. Gouarin S. Petitjean J.. (2005). Human Coronavirus NL63, France. Emerg. Infect. Dis. 11 (8), 1225–1229. doi: 10.3201/eid1108.050110 16102311 PMC3320486

[B77] van de Pol A. C. Boeijen J. A. Venekamp R. P. Platteel T. Damoiseaux R. Kortekaas M. F.. (2021). Impact of the COVID-19 Pandemic on Antibiotic Prescribing for Common Infections in The Netherlands: A Primary Care-Based Observational Cohort Study. Antibiotics (Basel). 10 (2), 196. doi: 10.3390/antibiotics10020196 33670657 PMC7922191

[B78] Wenzel R. P. Hendley J. O. Davies J. A. Gwaltney J. M. Jr. (1974). Coronavirus Infections in Military Recruits. Three-Year Study With Coronavirus Strains OC43 and 229E. Am. Rev. Respir. Dis. 109 (6), 621–624. doi: 10.1164/arrd.1974.109.6.621. 4365165

[B79] Wiertsema S. P. Chidlow G. R. Kirkham L. A. Corscadden K. J. Mowe E. N. Vijayasekaran S.. (2011). High Detection Rates of Nucleic Acids of a Wide Range of Respiratory Viruses in the Nasopharynx and the Middle Ear of Children With a History of Recurrent Acute Otitis Media. J. Med. Virol. 83 (11), 2008–2017. doi: 10.1002/jmv.22221 21915878 PMC7166877

[B80] Zumla A. Chan J. F. Azhar E. I. Hui D. S. Yuen K. Y. (2016). Coronaviruses - Drug Discovery and Therapeutic Options. Nat. Rev. Drug Discov. 15 (5), 327–347. doi: 10.1038/nrd.2015.37 26868298 PMC7097181

